# Prediction of distant metastasis in esophageal cancer using a radiomics–clinical model

**DOI:** 10.1186/s40001-022-00877-8

**Published:** 2022-12-03

**Authors:** Chao Zhu, Fengchun Mu, Songping Wang, Qingtao Qiu, Shuai Wang, Linlin Wang

**Affiliations:** 1 Department of Oncology, Qingdao Central Hospital Affiliated to Qingdao University, Qingdao, 266042 Shandong China; 2 Department of Radiation Oncology, Shandong Cancer Hospital and Institute, Shandong First Medical University and Shandong Academy of Medical Sciences, Jinan, 250117 Shandong China; 3 Department of Radiation Oncology, Affiliated Hospital of Weifang Medical University, Weifang, 261000 Shandong China

## Abstract

**Background:**

Distant metastasis, which occurs at a rate of 25% in patients with esophageal cancer (EC), has a poor prognosis, with previous studies reporting an overall survival of only 3–10 months. However, few studies have been conducted to predict distant metastasis in EC, owing to a dearth of reliable biomarkers. The purpose of this study was to develop and validate an accurate model for predicting distant metastasis in patients with EC.

**Methods:**

A total of 299 EC patients were enrolled and randomly assigned to a training cohort (*n* = 207) and a validation cohort (*n* = 92). Logistic univariate and multivariate regression analyses were used to identify clinical independent predictors and create a clinical nomogram. Radiomic features were extracted from contrast-enhanced computed tomography (CT) images taken prior to treatment, and least absolute shrinkage and selection operator (Lasso) regression was used to screen the associated features, which were then used to develop a radiomic signature. Based on the screened features, four machine learning algorithms were used to build radiomics models. The joint nomogram with radiomic signature and clinically independent risk factors was developed using the logical regression algorithm. All models were validated and compared by discrimination, calibration, reclassification, and clinical benefit.

**Results:**

Multivariable analyses revealed that age, N stage, and degree of pathological differentiation were independent predictors of distant metastasis, and a clinical nomogram incorporating these factors was established. A radiomic signature was developed by a set of sixteen features chosen from 851 radiomic features. The joint nomogram incorporating clinical factors and radiomic signature performed better [AUC(95% CI) 0.827(0.742–0.912)] than the clinical nomogram [AUC(95% CI) 0.731(0.626–0.836)] and radiomics predictive models [AUC(95% CI) 0.754(0.652–0.855), LR algorithms]. Calibration and decision curve analyses revealed that the radiomics–clinical nomogram outperformed the other models. In comparison with the clinical nomogram, the joint nomogram's NRI was 0.114 (95% CI 0.075–0.345), and its IDI was 0.071 (95% CI 0.030–0.112), *P* = 0.001.

**Conclusions:**

We developed and validated the first radiomics–clinical nomogram for distant metastasis in EC which may aid clinicians in identifying patients at high risk of distant metastasis.

**Supplementary Information:**

The online version contains supplementary material available at 10.1186/s40001-022-00877-8.

## Introduction

Esophageal cancer (EC) is a common digestive tract malignancy that accounts for 5.5% of cancer-related mortality; its incidence and mortality rates ranked seventh and sixth, respectively [1]. It was reported that approximately 20% of EC patients have distant metastasis at the time of diagnosis, and nearly half of the curatively treated patients develop distant metastasis within 5 years [2]. Distant metastasis is the external manifestation of cancer cells’ invasiveness [3]. As a result, patients with distant metastasis at the time of diagnosis have rapid disease progression and a poor prognosis, with a median survival time of only 3–5 months [4, 5]. Early identification of patients at high risk of distant metastasis and effective intervention will help to improve patients' prognoses. However, due to a scarcity of reliable biomarkers, there has been little research on predicting the risk of EC distant metastasis. Radiomics is a new field of study based on the assumption that extracted imaging data are the results of genetic and molecular mechanisms linked to genotypic and phenotypic characteristics [6, 7]. The fundamental goal of radiomics is to convert medical images into digital information, which includes basic descriptive parameters such as size, shape, intensity, and texture, as well as physiological parameters, such as contrast enhancement, diffusion characteristics, and tracer uptake [8]. These features provide information about cancer phenotype and tumor microenvironment, which are relatively independent and interconnected with traditional clinical and molecular characteristics, resulting in more accurate evidence-based medicine evidence [9, 10]. Radiomics is commonly utilized in oncology. It had been reported that radiomics can be used to predict the risk of colon cancer recurrence and prognosis [11]; exhibit the molecular characteristics of prostate cancer [12]; and be used for diagnosis, treatment follow-up, and identification of invasive disease [12, 13]. Breast cancer is one of the most active areas of radiomics study. MRI-based image features can be utilized to predict the metastasis of sentinel lymph nodes, the prognosis of early breast cancer, and the remission rate of neoadjuvant therapy [14, 15]. Radiomics based on CT, PET–CT, and MRI has the potential to improve the stratification for esophageal cancer and esophageal gastric junction cancer [16, 17]. Klaassen et al. reported that radiomics predicted the therapeutic effect of liver metastasis of gastroesophageal junction cancer with an AUC = 0.87 [18].

In this study, the clinicopathological predictors and radiomics were effectively integrated using a machine learning algorithm, and an accurate and reliable prediction model was developed by supervised learning to achieve accurate prediction of distant metastasis of EC, providing a powerful tool for individualized treatment of EC patients.

## Materials and methods

### Patients

This study collected esophageal cancer cases that were initially diagnosed in Shandong Cancer Hospital from January 2020 to October 2020, and it was approved by the ethics committee of Shandong Cancer Hospital (Ethics review approval No.: 2021003193), and informed consent was waived.

**Fig. 1:**
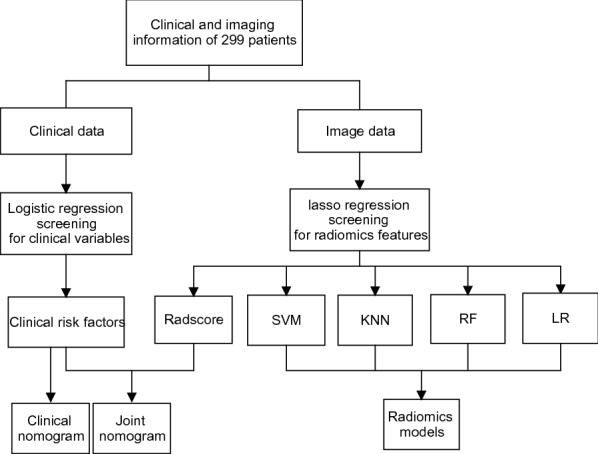
Flow chart of this study

All cases were histologically confirmed including Siewert type I esophageal gastric junction cancer, and no other primary malignant tumors. Before treatment, they were thoroughly examined for distant metastasis using whole-body ^18^FDG–PET–CT, or chest, abdomen, and pelvic enhanced CT, brain MRI, and radionuclide bone imaging. Cases with unconfirmed lesions, poor CT image quality, or significant artifacts were excluded. The random algorithm divided all cases into a training set and a validation set in a 7:3 ratio. Figure 1 depicts a research flowchart.

### Data and images collection

By consulting the medical record system, clinical data such as gender, age, histology, TNM stage, tumor location, length, histological grade, and metastatic site were obtained. TNM staging was performed in accordance with the eighth edition of the American Joint Committee on Cancer (AJCC) staging of esophageal and gastroesophageal junction tumors. All CT images were acquired using a picture archiving and communication system (PACS) and saved in digital imaging and communications in medicine (DICOM) format.

The CT equipment parameters were Philips brilliance iCT 128 rows, tube voltage 120 kV, tube current 368 mAs, layer thickness 5 mm, pixel spacing (0.78125, 0.78125), and image matrix 512 × 512.

### Image segmentation and radiomic features extraction

For image segmentation, 3D Slicer (version: 4.10.2), an open source software platform for medical image processing and visualization, was used. The arterial phase was chosen for image segmentation, because it is more conducive to displaying esophageal tumors [19]. Primary tumors, defined as lesions with esophageal wall thickening > 5 mm or lumen occlusion diameter > 10 mm and excluding intraluminal gas and oral contrast agent, were included in the region of interest (ROI). Normal structures and metastatic lymph nodes were left out. Window width 500 and window level 40 were the parameters for ROIs. A doctor with 10 years of experience in the radiotherapy department manually completed ROIs, which were then reviewed by a radiologists. The patient's clinical information was unknown to both doctors. Pyradiosity, an open source Python package that can be found at https://pyradiomics.readthedocs.io/en/latest/, was used to extract all of the features.

### Radiomic feature selection and signature construction

The values of the extracted radiomic features were normalized using the formula (x-Min)/(Max–Min), resulting in values ranging from 0 to 1. The radiomic features most associated with esophageal cancer distant metastasis were screened using the least absolute shrinkage and selection operator regression (Lasso) and logistic regression algorithms. The Pearson correlation test was used to rule out multicollinearity, and any correlation coefficient with an absolute value greater than 0.9 was considered multicollinear [20].

The sum of all filtered eigenvalues multiplied by the corresponding coefficients equaled the radiomic signature (radscore). radscore = *β*_1_*X*_1_ + *β*_2_*X*_2_ + …*β*_*n*_*X*_*n*_, where radscore was the radiomic signature, *β*_*n*_ was the coefficient, and *X*_*n*_ was the eigenvalue [21].

**Fig. 2:**
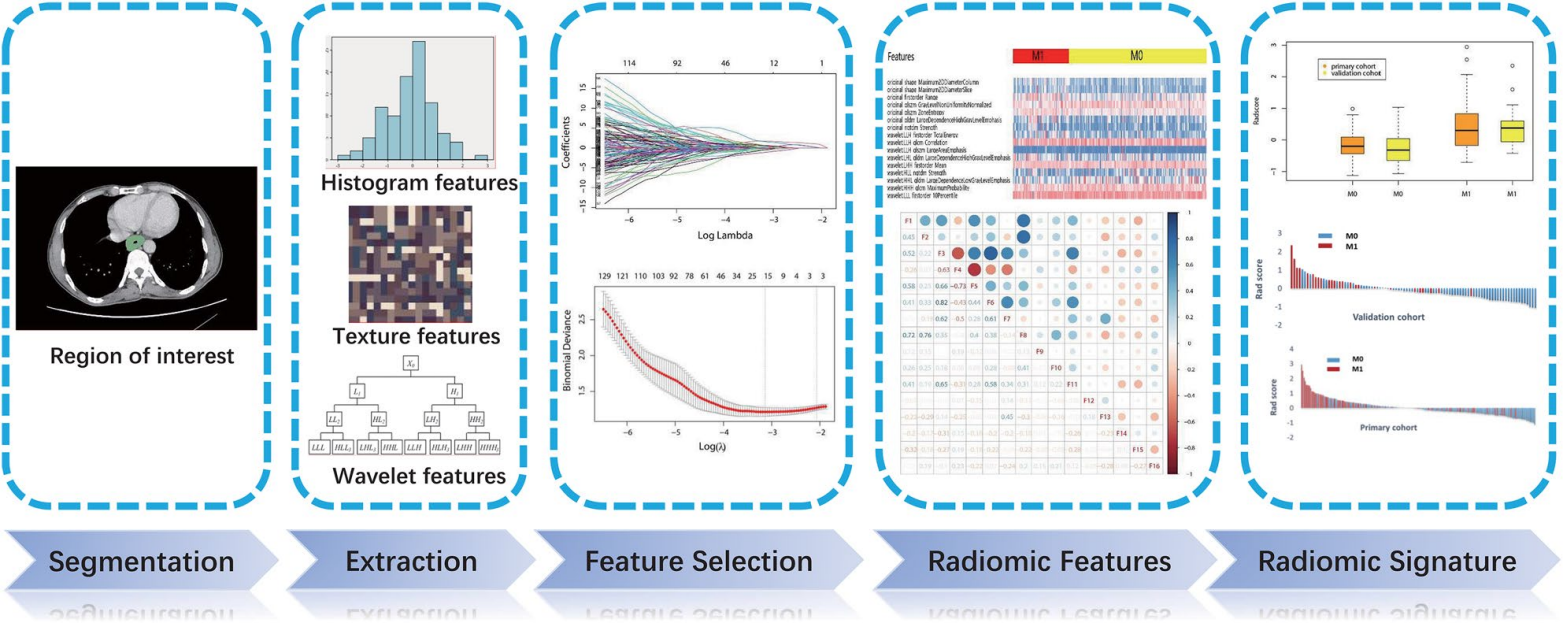
Procedure for creating a radiomics signature. From the 851 radiomic features extracted from CT-ROIs, the Lasso algorithm selected 16 features that had the best correlation with distant metastasis and constructed the radiomic signature. The box chart and bar plot revealed that there was no significant difference between the training and validation sets, but there was a difference between patients with and without metastasis

The Wilcoxon rank sum (Mann Whitney) test was used to assess the consistency of radcore in the training and validation sets. Figure 2 depicts the process of creating a radiomic signature.

### Clinical model construction

The clinical independent predictors were identified by logistic univariate and multivariate regression analyses, and a clinical nomogram was created using the independent predictors.

### Radiomics model construction

The support vector machine (SVM), random forest (RF), k nearest neighbor (KNN), and logistic regression (LR) machine learning algorithms were used to build radiomics models based on selected radiomic features for comparison with clinical and radiomics–clinical models.

### Radiomics–clinical model construction

Based on the training samples, clinical predictors with statistical significance in univariate regression analysis and radiomic signature (radscore) were included in logistic multivariate regression analysis, and variables with *P* < 0.05 were retained to build a joint prediction model.

### Performance and comparison

Five measures recommended by Steyerberg et al. [22] were used in this study to evaluate the performance of prediction models:

i. The receiver operating characteristic curve (ROC) and area under curve (AUC) were used to assess discrimination, and the AUC of the two models was compared using Delong test.
ii. Calibration curves were used to confirm calibration.
iii. Akaike information criterion (AIC) was used to present the complexity and goodness of fit.
iv. Reclassification was evaluated and compared using net reclassification improvement (NRI) and integrated discrimination improvement (IDI).
v. Decision curve (DCA) was used to reflect each model’s clinical benefit.

### Statistical analysis

Stata 15.0 (Stata Corp, www.stata.com) was used to conduct a statistical analysis of baseline characteristics between groups. For categorical variables, the Chi square test was used, and for continuous variables, the Mann–Whitney test was used. Statistical significance was defined as a *P* value of less than 0.05. To screen radiomic features, construct, and verify prediction models, R software (version3.5.1, https://www.r-project.org/) was used. In the table below, the packages used in R software were listed.

**Table:** 

Algorithm	Package	Version
Lasso	glmnet	4.0–1
Logistic, Nomogram, Calibration curve	rms	6.0–1
KNN	class, kknn	7.3–17, 1.3.1
RF	randomForest	4.6–14
SVM	e1071	1.7–4
ROC/AUC	ROCR, pROC	1.0–11, 1.16.2
NRI/IDI	PredictABEL, nricens	1.2–4, 1.6
Correlation test	corrplot	0.84
DCA curve	rmda	1.6

## Results

### Patients’ characteristics

A total of 299 patients with newly diagnosed esophageal cancer were included in the study, with 102 (34%) undergoing surgery and 53 (18%) undergoing ^18^FDG–PET–CT examination. Patients with distant metastasis made up 34% (102) and patients without distant metastasis made up 66% (197) of the total. The most common metastatic sites were non-regional lymph nodes in 86 cases (64%), lung in 19 cases (14%), liver in 20 cases (15%), and bone, adrenal gland, peritoneum, and pericardium in 20 cases (15%). Sixty-six cases of single organ metastasis (64.7%) and 36 cases of multiple organ metastasis (35.3%) were reported (non-regional lymph nodes were calculated as one organ).

According to the 7:3 ratio, all patients were randomly assigned to the training and validation groups. In the training set, 76 patients had distant metastasis and 131 did not; in the validation set, 26 patients had distant metastasis and 66 did not. Gender, age, stage, length, and location were not significantly different in the training and validation groups (*P* < 0.05). The baseline characteristics of patients are presented in Table 1.

**Table 1:** Clinical characteristics of patients included in the analysis

Characteristics	Primary cohort				Validation cohort			
	M_0_^a^	M_1_^b^	χ^2^	*P*	M_0_^a^	M_1_^b^	χ^2^	*P*
Subjects	131	76			66	26		
Age								
≥ 70	49(37.40%)	18(23.68%)			25(37.88%)	8(30.77%)		
69–60	56(42.75%)	26(34.21%)			30(45.45%)	10(38.46%)		
< 60	26(19.85%)	32(42.11%)	12.190	0.002	11(16.67%)	8(30.77%)	2.269	0.322
Length								
≤ 4 cm	40(30.53%)	18(23.68%)			18(27.27%)	9(34.62%)		
> 4 cm	91(69.47%)	58(76.32%)	1.120	0.290	48(72.73%)	17(65.38%)	0.485	0.486
Gender								
Female	30(22.90%)	11(14.47%)			8(12.12%)	6(23.08%)		
Male	101(77.10%)	65(85.53%)	2.150	0.143	58(87.88%)	20(76.92%)	1.735	0.188
Pathological grading								
G_x_	73(55.73%)	41(53.95%)			31(46.97%)	13(50.00%)		
G_1_/G_2_	41(31.30%)	4(5.26%)			25(37.88%)	5(19.23%)		
G_3_	17(12.98%)	31(40.79%)	31.069	0.000	10(15.15%)	8(30.77%)	4.350	0.114
Stage_T								
T_1_/T_2_	14(10.69%)	3(3.95%)			11(16.67%)	1(3.85%)		
T_3_/T_4_	117(89.31%)	73(96.05%)	2.898	0.089	55(83.33%)	25(96.15%)	2.703	0.100
Stage_N								
N_0_	52(39.69%)	4(5.26%)			27(40.91%)	2(7.69%)		
N_1_	44(33.59%)	27(35.53%)			22(33.33%)	12(46.15%)		
N_3_/N_2_	35(26.72%)	45(59.21%)	34.269	0.000	17(25.76%)	12(46.15%)	9.820	0.007
Histology								
Squamous	117(89.31%)	63(82.89%)	1.747	0.186	61(92.42%)	21(80.77%)	2.615	0.106
Others	14(10.69%)	13(17.11%)			5(7.58%)	5(19.23%)		
Tumor site								
Upper	19(14.50%)	12(15.79%)			3(4.55%)	6(23.08%)		
Middle	51(38.93%)	28(36.84%)			22(33.33%)	4(15.38%)		
Lower	61(46.56%)	36(47.37%)	0.115	0.944	41(62.12%)	16(61.54%)	8.675	0.013

^a^Patients without distant metastasis

^b^Patients with distant metastasis

### Radiomic features selection and radiomic signature

From each ROI, 851 radiomic features were extracted, and the Lasso regression algorithm screened 16 features that were most closely related to the presence of malignant esophageal fistula (Additional file 1: Appendix S1). Pearson correlation coefficient had a maximum value of 0.82 indicating that there were no seriously associated variables (absolute value of correlation coefficient > 0.9). The filtered radiomic feature set, related coefficients, and radiomic signature are depicted in Fig. 2. The radiomic signature (radscore) calculation formula was as follows:

$$\begin{aligned} {\text{Radscore }} = \, & \,{\text{original}}\_{\text{shape}}\_{\text{Maximum2DDiameterColumn}} \times {\text{1}}.{\text{75}}0{\text{4498}}0 \\ & + \,{\text{original}}\_{\text{shape}}\_{\text{Maximum2DDiameterSlice}} \times 0.{\text{488878}}0{\text{3}} \\ & + \,{\text{original}}\_{\text{firstorder}}\_{\text{Range}} \times 0.0{\text{1331597}} \\ & + \,{\text{original}}\_{\text{glszm}}\_{\text{GrayLevelNonUniformityNormalized}} \times - \,0.{\text{5259}}0{\text{144}} \\ & + \,{\text{original}}\_{\text{glszm}}\_{\text{ZoneEntropy}} \times 0.{\text{369258}}0{\text{2}} \\ & + \,{\text{original}}\_{\text{gldm}}\_{\text{LargeDependenceHighGrayLevelEmphasis}} \times 0.{\text{64118898}} \\ & + \,{\text{original}}\_{\text{ngtdm}}\_{\text{Strength}} \times 0.0{\text{8979331}} \\ & + \,{\text{wavelet}}.{\text{LLH}}\_{\text{firstorder}}\_{\text{TotalEnergy}} \times {\text{1}}.{\text{52259472}} \\ & + \,{\text{wavelet}}.{\text{LLH}}\_{\text{glcm}}\_{\text{Correlation}} \times - \,0.{\text{1}}00{\text{57724}} \\ & + \,{\text{wavelet}}.{\text{LLH}}\_{\text{glszm}}\_{\text{LargeAreaEmphasis}} \times - \,0.{\text{14142835}} \\ & + \,{\text{wavelet}}.{\text{LHL}}\_{\text{gldm}}\_{\text{LargeDependenceHighGrayLevelEmphasis}} \times 0.{\text{11553639}} \\ & + \,{\text{wavelet}}.{\text{LHH}}\_{\text{firstorder}}\_{\text{Mean}} \times 0.{\text{48958615}} \\ & + \,{\text{wavelet}}.{\text{HLL}}\_{\text{ngtdm}}\_{\text{Strength}} \times 0.{\text{53664977}} \\ & + \,{\text{wavelet}}.{\text{HHL}}\_{\text{gldm}}\_{\text{LargeDependenceLowGrayLevelEmphasis}} \times - \,0.{\text{16999177}} \\ & + \,{\text{wavelet}}.{\text{HHH}}\_{\text{glcm}}\_{\text{MaximumProbability}} \times - \,{\text{1}}.0{\text{8283494}} \\ & + \,{\text{wavelet}}.{\text{LLL}}\_{\text{firstorder}}\_{\text{1}}0{\text{Percentile}} \times - \,0.{\text{44247459}}{\text{.}} \\ \end{aligned}$$

In the training set, patients with distant metastasis had a radscore of 0.399 ± 0.736, while those without had a radscore of − 0.1781 ± 0.420, *z *= − 5.857, *P* = 0.000; in the validation set, patients with distant metastasis had a radscore of 0.411 ± 0.630, while those without had a radscore of − 0.260 ± 0.457, *z* = − 4.856, *P* = 0.000. There was no difference in radscore between non-distant metastasis patients (*z* = − 1.506, *P* = 0.1319) and distant metastasis patients (*z* = − 0.392, *P* = 0.6953) in the training set and the validation set.

### Development and validation of the clinical model

The clinical suspicious risk factors were all filtered by logistic univariate regression analysis. The findings revealed that there were significant differences in age, N stage, and degree of pathological differentiation (Table 2). Multivariate analysis took into account the factors listed above, and age, N stage, pathological differentiation were the independent risk factors (*P* < 0.05).

**Table 2:** Univariate regression analysis and multivariate regression analysis

Characteristics	Univariate regression	*P*	Multivariate regression	*P*
	OR(95%CI)		OR(95%CI)	
Age				
≥ 70	Reference		Reference	
69–60	1.264 (0.622–2.605)	0.520	1.356 (0.596–3.144)	0.470
< 60	3.350 (1.604–7.197)	0.002	2.911 (1.223–7.147)	0.017
Length				
≤ 4 cm	Reference			
> 4 cm	1.416 (0.750–2.748)	0.291		
Gender				
Female	Reference			
Male	1.755 (0.843–3.886)	0.146		
Grade				
G_x_	Reference			
G_1_/G_2_	0.174 (0.05–0.469)	0.002	0.216 (0.058–0.633)	0.010
G_3_	3.247 (1.623–6.680)	0.001	3.042 (1.394–6.901)	0.006
Stage_T				
T_1_/T_2_	Reference			
T_3_/T_4_	2.912 (0.912–12.941)	0.102		
Stage_N				
N_0_	Reference			
N_1_	7.977 (2.854–28.542)	0.000	6.126 (2.067–22.783)	0.002
N_3_/N_2_	16.714 (6.112–59.182)	0.000	13.498 (4.698–49.428)	0.000
Histology				
Squamous	Reference			
Others	1.724 (0.756–3.914)	0.190		
Location				
Upper	Reference			
Middle	0.869 (0.371–2.084)	0.749		
Lower	0.934 (0.410–2.189)	0.873		
Radscore	7.133 (3.735–14.879)	0.000	5.214 (2.369–12.885)	0.000

OR, odds ratio; CI, confidence interval

**Fig. 3:**
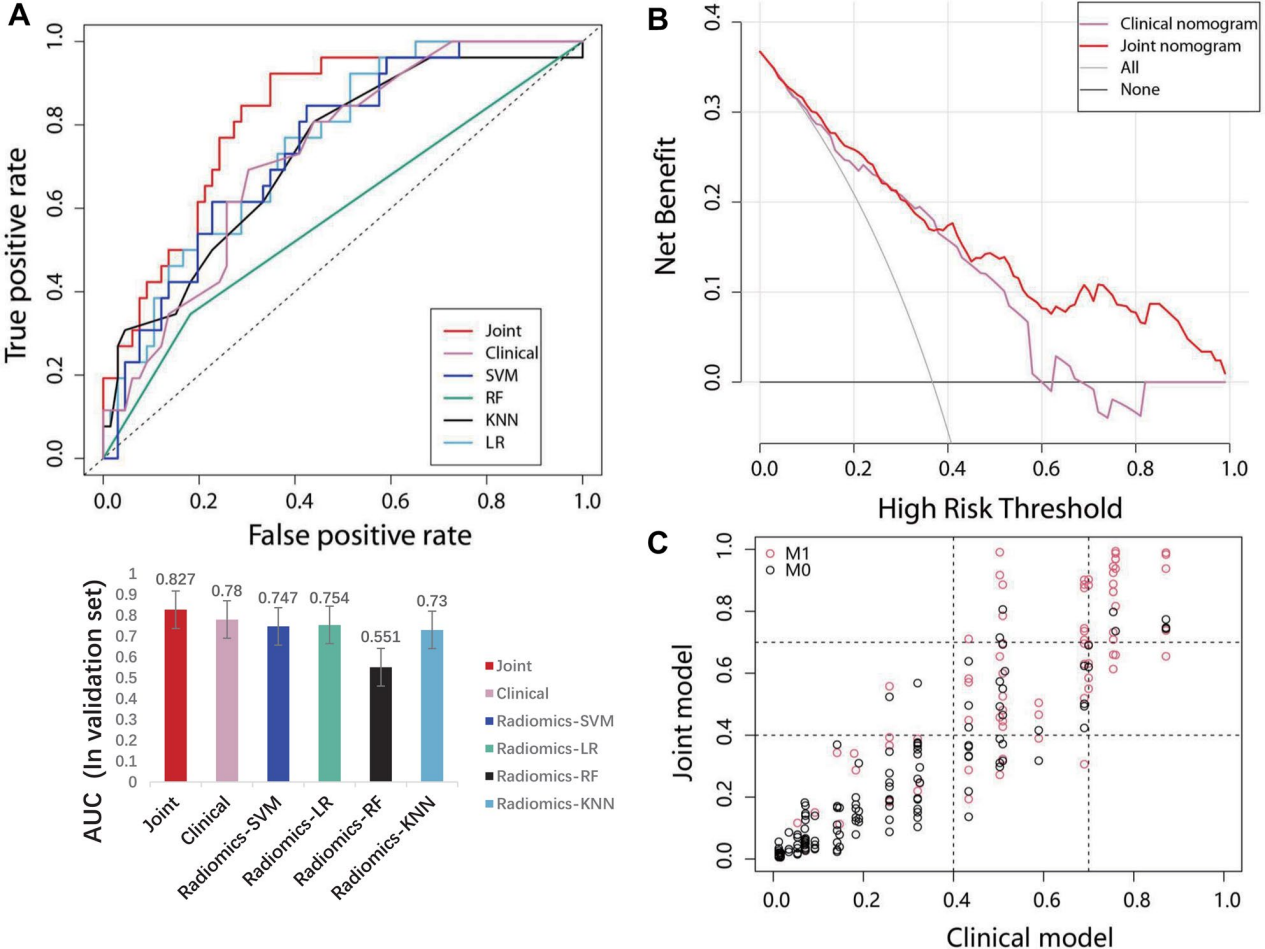
**A** Nomogram developed by clinical predictors. **B** ROC curve and **C** Calibration curve plotted for clinical prediction model. **D** radiomics–clinical nomogram, **E** ROC curve and **F** calibration curve plotted for radiomics–clinical prediction model

We built a clinical prediction model with independent risk factors to compare to the radiomics model and radiomics–clinical model that we subsequently developed. The AUC was 0.731 (95% CI 0.626–0.836) in validation set, 0.82 (95% CI 0.773–0.886) in training set, and the AIC was 215.9. Figure 3 depicts the clinical prediction model's nomogram, ROC curve and calibration curve.

### Development and validation of the radiomics models

Support vector machines (SVM), K nearest neighbor (KNN), random forest (RF) and logistic regression (LR) were used to create radiomics models, respectively. Table 3 shows the performance details of the models. The LR algorithm model's discrimination was superior to the other three.

**Table 3:** Performance of radiomics models

Algorithms	AUC(95%CI) in validation set
Support vector machines (SVM)	0.747 (0.642–0.851)
Random forest (RF)	0.551 (0.478–0.687)
K nearest neighbor (KNN)	0.730 (0.617–0.843)
Logistic regression (LR)	0.754 (0.652–0.855)

### Development and validation of the radiomics–clinical model

There was a significant difference in radscores between patients with distant metastasis and those without (*P* = 0.000) according to multivariate regression analysis. To create a joint nomogram model, radcore was fitted with age, degree of pathological differentiation, and N-stage using a logistic regression algorithm. In the validation set, the AUC was 0.827 (95%CI 0.742–0.912), in the training set, 0.857 (95% CI 0.806–0.908), indicating excellent discrimination. Predicted values were in good agreement with observed results, according to the calibration curve (Fig. 3).

### Comparison of models

**Fig. 4:**
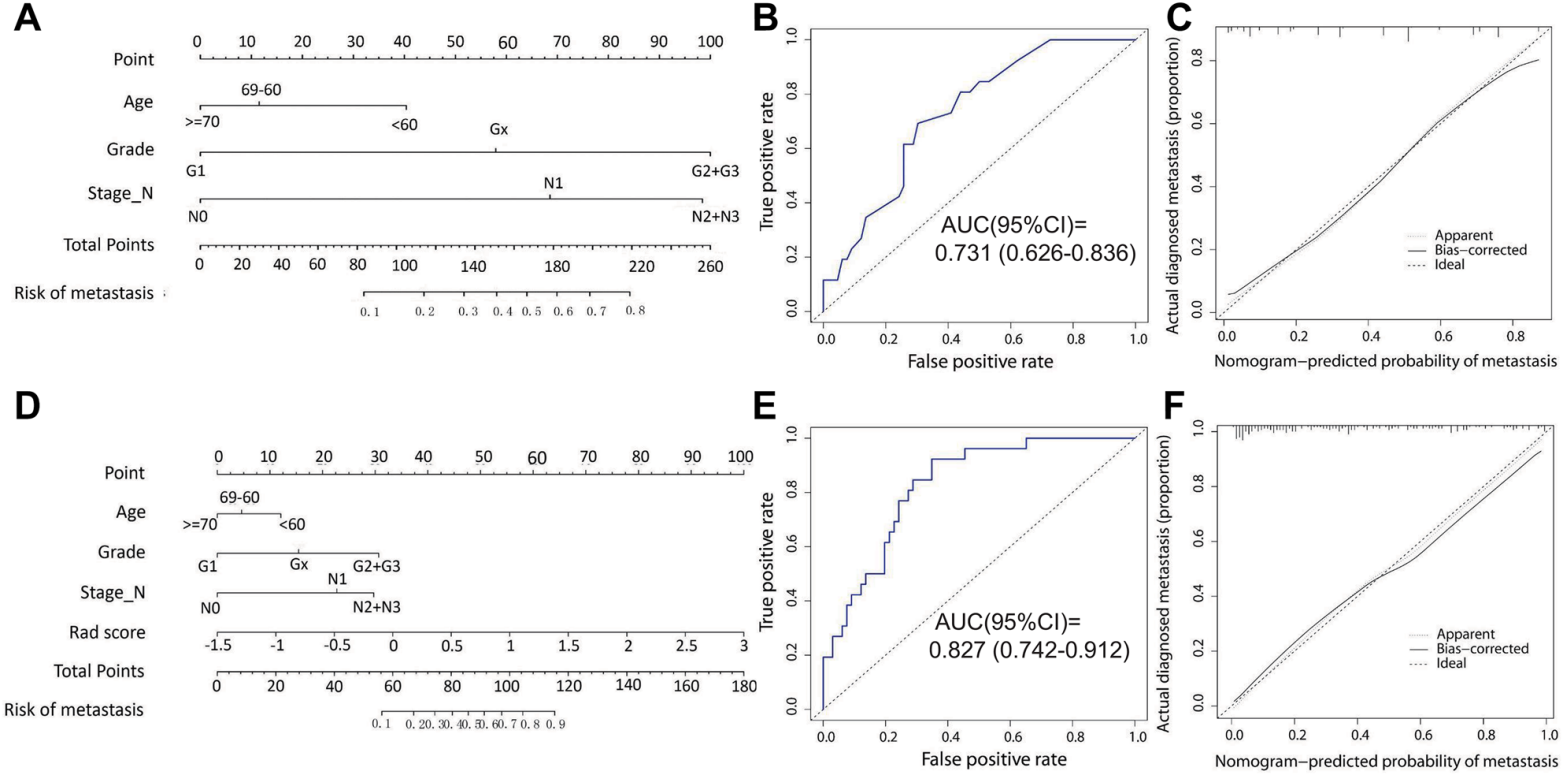
Model's performance was compared using the **A** ROC curve (discrimination), **B** DCA curve (clinical benefit), and **C** NRI plot (reclassification). The results revealed that the radiomics–clinical (joint) model outperformed the other models

Figure 4A shows the performance comparison of clinical model, radiomics model, and radiomics–clinical model. The Delong test revealed that the radiomics–clinical model discriminated better than the radiomics and clinical models (*P* < 0.05). The net benefit of the radiomics–clinical model was better than the clinical model under each threshold probability, as shown by the decision curve (Fig. 4B). The NRI of the radiomics–clinical model was 0.113 (95% CI 0.075–0.345) (1000 iterations) (Fig. 4C), and the IDI was 0.071 (95% CI 0.0301–0.1122), *P* = 0.00068, when compared to the clinical model.

## Discussion

Based on clinical factors and radiomics, this study developed and validated a nomogram prediction model for distant metastasis of esophageal cancer with high discrimination and robustness.

The main clinical predictors of distant metastasis were age, pathological differentiation, and N stage. The clinical prediction model performed well, with an AUC of 0.731, which was consistent with our previous research [23]. The radiomics–clinical model was more accurate after the addition of radiomics, with an AUC = 0.827, demonstrating that radiomics can supplement clinical risk factors in the prediction of distant metastasis of EC.

Radiomics played an important role in predicting tumor metastasis, because medical images can show molecular phenotypes of tumors from a macro-perspective [24, 25]. The goal of radiomics is to convert images into data that can be mined, extracted, and analyzed [26]. The radiomic features of primary lesions can help predict lymph node metastasis of esophageal cancer. Qu et al. screened texture features from MRI images, concentrating on length, shape, gray-level co-occurrence matrix (GLCM), and gray-level run length (GLRL). The radiomic signature created by these features can accurately determine whether esophageal cancer patients have lymph node metastasis with an AUC = 0.821, (95% CI 0.7042–0.9376) [27]. In a retrospective study of 230 patients with esophageal cancer, Zhang et al. discovered that CT-based radiomics can be used to predict lymph node metastasis, which is more accurate than simply using lymph node size as the judgment standard [28]. Radiomics of esophageal cancer can be used as a biomarker to predict radiotherapy and chemotherapy efficacy [29–31]. The application of radiomics in the treatment of prostate, lung, and breast cancer has also been studied [12–15]. According to Tunali et al., radiomic features capture biological and pathological information, which has been shown to provide rapid and noninvasive biomarkers for lung cancer risk prediction, diagnosis, prognosis, and tumor biology [32].

In this study, the Lasso-logistic regression algorithm was used to screen high-dimensional radiomic features, and 16 features associated with distant metastasis and without multicollinearity were chosen. The majority of them were texture features or texture features after wavelet transformation, such as the gray-level co-occurrence matrix (GLCM), gray-level size zone matrix (GLSZM), gray-level dependence matrix (GLDM), and neighborhood gray tone difference matrix (NGTDM). Wu et al. filtered 10 radiomic features from 6140 to distinguish early esophageal cancer from advanced esophageal cancer. These features were primarily found in GLCM, GLRLM, GLSZM, and NGTDM [33], which were consistent with our findings. KNN, RF, SVM, and LR algorithms were used to build models based on these selected features to evaluate the maximum efficiency of radiomics in predicting distant metastasis of esophageal cancer and select the best algorithm for fitting the features. The results demonstrated that the prediction ability of radiomics models constructed by machine learning algorithms other than RF was comparable to that of clinical factors for the prediction of esophageal cancer distant metastasis and can be used as a marker to predict EC distant metastasis on its own.

A multi-feature-based radiomics signature can provide more information than a single parameter [21]. The combination of radiomic signature and clinicopathological factors through machine learning can optimize the performance of prediction models [34, 35]. In this study, the efficiency of the radiomics–clinical model (AUC = 0.827) was significantly higher than that of the clinical model (AUC = 0.731) and the radiomics model (AUC = 0.754) (Delong test, *P* < 0.05). The goodness of fit was presented by Akaike information criterion (AIC). The AIC of the radiomics–clinical model was lower than that of the clinical and radiomics models, indicating a better fitness. The DCA curve, NRI, and IDI were also used to compare the performance of various models [22]. The ROC curve compares prediction accuracy only from the standpoint of discrimination, whereas the DCA curve displays the potential risks and benefits of false negative and false positive [36]. NRI is defined as the difference in the number of correct classifications between two classifiers, which can be understood as the difference between the sums of the sensitivity and specificity of two classifiers [37]. IDI is similar to NRI in that it refers to the quantification of the prediction probability gap [38]. These two indicators are better suited for model comparison [38].

This study's ROIs were based on the arterial phase image of enhanced CT, that was, 30–35 s after the injection of enhancer, drawing lessons from Umeoka’s study. According to this study, the difference between esophageal tumor and normal esophageal wall in the second arterial phase (delayed 35 s) is significantly greater than in the first arterial phase (delayed 5 s) and venous phase (delayed 65 s) [19].

The following are the benefits of this research: for starters, this is the first radiomics–clinical prediction model for EC distant metastasis. Second, all ROIs outlined in the study included the entire esophageal tumor rather than partial tomographic images reported in previous studies, which can better present biological properties of whole tumors and have better repeatability. Third, ROIs were manually outlined to avoid the lumen area and minimize the impact of lumen contents on ROIs. Furthermore, the model was presented as a nomogram, which can more intuitively show the impact of various parameters on the outcome and is more convenient for clinical application.

The current study has some limitations. First, despite the model's strong performance, it lacked the external validation sets necessary to back up its generalization. For further validation, it is, therefore, necessary to include patients from other centers in the follow-up study. Second, because this was a retrospective study, selection bias was unavoidable, even though we used strict inclusion criteria. Third, because the goal of this study was to develop a reliable metastasis prediction model, the mechanism was not thoroughly investigated, necessitating more thorough investigations.

## Conclusions

Radiomics of arterial phase CT images prior to treatment can be used to predict distant EC metastasis. The radiomics–clinical model can more accurately predict EC distant metastasis, which is useful for early discrimination of patients at high risk of distant metastasis.

## Supplementary Information

**Additional file 1: Appendix S1.** Radiomic features.

## Data Availability

Data and materials were available.

## Abbreviations

EC: Esophageal cancer
CT: Computed tomography
Lasso: Least absolute shrinkage and selection operator
AJCC: American Joint Committee on Cancer
PACS: Picture archiving and communication
DICOM: Digital imaging and communications in medicine
ROI: Region of interest
SVM: Support vector machine
RF: Random forest
KNN: K nearest neighbor
LR: Logistic regression
ROC: Receiver operating characteristic curve
AUC: Area under curve
AIC: Akaike information criterion
NRI: Net reclassification improvement
IDI: Integrated discrimination improvement
DCA: Decision curve
GLCM: Gray-level co-occurrence matrix
GLRL: Gray-level run length
