# Supplementary material for: Prediction of distant metastasis in esophageal cancer using a radiomics–clinical model
Source: Eur J Med Res. 2022 Dec 3;27:272. doi: 10.1186/s40001-022-00877-8 (PMC9719117; doi:10.1186/s40001-022-00877-8)
Supplement: Supplementary file 1 — Additional file 1: Appendix S1. Radiomic features. [file 40001_2022_877_MOESM1_ESM.docx]

**Radiomics Features**

**Radiomic Features Extraction**

ALL extracted radiomics features can be divided into 4 categories. The ‘shape’ category including 14 features, which represents the shape and size of region of interest (ROIs) in 2D and 3D spaces. The ‘first-order’ represents statistical eigenvalues of voxel intensity, including mean, maximum, and minimum values. Textural features including Glcm(GrayLevelCooccurenceMatrix), Glrlm(GrayLevelRunLengthMatrix), Glszm(GrayLevelSizeZoneMatrix), Gldm(GrayLevelDependenceMatrix), Ngtdm(NeighbouringGrayToneDifferenceMatrix) were calculated from several statistical matrices and described the arrangement of voxel intensity within ROIs. Wavelet-based features were defined as first-order and texture features calculated from eight wavelet decompositions of the original CT image. Therefore, the total number of radiomic features could be calculated as 14+(18+24+16+16+14+5) +(18+24+16+16+14+5)×8=851.

**Table S1. Features used in this study.**

| **Categories** | **N** | **Features** |
| --- | --- | --- |
| Shape | 14 | Elongation  Flatness  LeastAxisLength  MajorAxisLength  Maximum2DDiameterColumn  Maximum2DDiameterRow  Maximum2DDiameterSlice  Maximum3DDiameter  MeshVolume  MinorAxisLength  Sphericity  SurfaceArea  SurfaceVolumeRatio  VoxelVolume |
| firstorder | 18 | 10Percentile  90Percentile  Energy  Entropy  InterquartileRange  Kurtosis  Maximum  MeanAbsoluteDeviation  Mean  Median  Minimum  Range  RobustMeanAbsoluteDeviation  RootMeanSquared  Skewness  TotalEnergy  Uniformity  Variance |
| Glcm(GrayLevelCooccurenceMatrix) | 24 | Autocorrelation  ClusterProminence  ClusterShade  ClusterTendency  Contrast  Correlation  DifferenceAverage  DifferenceEntropy  DifferenceVariance  Id  Idm  Idmn  Idn  Imc1  Imc2  InverseVariance  JointAverage  JointEnergy  JointEntropy  MCC  MaximumProbability  SumAverage  SumEntropy  SumSquares |
| Glrlm(GrayLevelRunLengthMatrix) | 16 | GrayLevelNonUniformity  GrayLevelNonUniformityNormalized  GrayLevelVariance  HighGrayLevelRunEmphasis  LongRunEmphasis  LongRunHighGrayLevelEmphasis  LongRunLowGrayLevelEmphasis  LowGrayLevelRunEmphasis  RunEntropy  RunLengthNonUniformity  RunLengthNonUniformityNormalized  RunPercentage  RunVariance  ShortRunEmphasis  ShortRunHighGrayLevelEmphasis  ShortRunLowGrayLevelEmphasis |
| Glszm(GrayLevelSizeZoneMatrix) | 16 | GrayLevelNonUniformity  GrayLevelNonUniformityNormalized  GrayLevelVariance  HighGrayLevelZoneEmphasis  LargeAreaEmphasis  LargeAreaHighGrayLevelEmphasis  LargeAreaLowGrayLevelEmphasis  LowGrayLevelZoneEmphasis  SizeZoneNonUniformity  SizeZoneNonUniformityNormalized  SmallAreaEmphasis  SmallAreaHighGrayLevelEmphasis  SmallAreaLowGrayLevelEmphasis  ZoneEntropy  ZonePercentage  ZoneVariance |
| Gldm(GrayLevelDependenceMatrix) | 14 | DependenceEntropy  DependenceNonUniformity  DependenceNonUniformityNormalized  DependenceVariance  GrayLevelNonUniformity  GrayLevelVariance  HighGrayLevelEmphasis  LargeDependenceEmphasis  LargeDependenceHighGrayLevelEmphasis  LargeDependenceLowGrayLevelEmphasis  LowGrayLevelEmphasis  SmallDependenceEmphasis  SmallDependenceHighGrayLevelEmphasis  SmallDependenceLowGrayLevelEmphasis |
| Ngtdm(NeighbouringGrayToneDifferenceMatrix) | 5 | Busyness  Coarseness  Complexity  Contrast  Strength |
